# Metformin against Cancer Stem Cells through the Modulation of Energy Metabolism: Special Considerations on Ovarian Cancer

**DOI:** 10.1155/2014/132702

**Published:** 2014-06-24

**Authors:** Tae Hun Kim, Dong Hoon Suh, Mi-Kyung Kim, Yong Sang Song

**Affiliations:** ^1^Department of Obstetrics and Gynecology, Korean Cancer Center Hospital, Korea Institute of Radiological and Medical Sciences, Seoul 139-706, Republic of Korea; ^2^Department of Obstetrics and Gynecology, Seoul National University Bundang Hospital, Seongnam 463-707, Republic of Korea; ^3^Biomedical Science Project, Brain Korea 21 Program for Leading Universities & Students, Seoul National University, Seoul 110-799, Republic of Korea; ^4^Department of Obstetrics and Gynecology, Seoul National University College of Medicine, Seoul 110-744, Republic of Korea; ^5^Cancer Research Institute, Seoul National University College of Medicine, Seoul 110-799, Republic of Korea; ^6^Major in Biomodulation, World Class University, Seoul National University, Seoul 151-921, Republic of Korea

## Abstract

Ovarian cancer is the most lethal gynecologic malignancy among women worldwide and is presumed to result from the presence of ovarian cancer stem cells. To overcome the limitation of current anticancer agents, another anticancer strategy is necessary to effectively target cancer stem cells in ovarian cancer. In many types of malignancies, including ovarian cancer, metformin, one of the most popular antidiabetic drugs, has been demonstrated to exhibit chemopreventive and anticancer efficacy with respect to incidence and overall survival rates. Thus, the metabolic reprogramming of cancer and cancer stem cells driven by genetic alterations during carcinogenesis and cancer progression could be therapeutically targeted. In this review, the potential efficacy and anticancer mechanisms of metformin against ovarian cancer stem cells will be discussed.

## 1. Introduction

Epithelial ovarian cancer (EOC) is the most lethal gynecologic malignancy and frequently presents with peritoneal carcinomatosis [1]. Approximately 60% to 70% of cases are diagnosed in advanced stages, and the five-year survival rate is 30% for advanced-stage ovarian cancer [2]. Although cytoreductive surgery and adjuvant chemotherapy are effective in extirpating tumor mass, approximately 80% of patients with advanced-stage EOC experience recurrence, after which chemotherapy is no longer as effective as it was previously [3]. Additionally, EOC exhibits high intratumoral heterogeneity at both the genotypic and phenotypic levels, with diverse biologic consequences [4]. The cancer stem cell (CSC) model is one of the emerging mechanisms of chemoresistance and intratumoral heterogeneity in EOC. CSCs, also called tumor initiating cells (TICs), are a subset of cancer cells within each tumor that exhibit the ability to induce tumors when transplanted into immune-deficient mice. CSCs are thought to constitute a small subset of cells within a tumor that initiate both the primary disease and its recurrence because of their capacity for self-renewal and inherent chemoresistance [5]. CSCs also possess properties of self-renewal as well as the ability to undergo serial passages in vitro and in vivo due to unlimited division potential [6].

Alterations in cellular bioenergetics are an emerging hallmark of cancer. The shift from oxidative phosphorylation to aerobic glycolysis (i.e., the Warburg effect) is the best-characterized metabolic phenotype of cancer [7]. Recent studies demonstrate that aerobic glycolysis is a characteristic of proliferating cells as well as the metabolic phenotype used by pluripotent cells, including CSCs [8]. Metformin is an oral antidiabetic drug in the biguanide class and the first-line drug of choice for the treatment of type 2 diabetes. Considering the association between CSCs and metabolic reprogramming, it is intriguing that metformin exhibits an anticancer effect, especially to CSCs [9–13]. However, the molecular mechanism by which metformin inhibits CSCs is unclear. This review will discuss the potential anticancer effect of metformin based on the hypotheses of CSC in the context of metabolic reprogramming in ovarian cancer.

## 2. Cancer Stem Cells as a Cause of Chemoresistance in Ovarian Cancer

The current first-line chemotherapy for EOC patients is a combination of carboplatin and paclitaxel [14]. Most relapses are likely the result of sparing of ovarian CSCs. Resistance to conventional chemotherapy has also been suggested to be a unique property of CSCs. Decreased responsiveness to chemotherapy might be due in part to the slow proliferation rates of CSCs, given that conventional cytotoxic drugs mainly target highly proliferative cells [15]. High expression of ATP-binding cassette drug transporters and antiapoptotic proteins, the ability to protect cells from DNA damage, and efficient DNA repair have been suggested to be a cause of chemoresistance of CSCs [16]. In addition, ovarian CSCs are resistant to tumor necrosis factor  *α*-mediated apoptosis [5].

Several studies have demonstrated that chemotherapy-treated residual tumors are enriched in cells with a CSC phenotype, whereas cells lacking these characteristics are eliminated [17–20]. Steg et al. reported that a proportion of ovarian CSCs seem to be increased after chemotherapy [20]. Primary samples were composed of low densities of CSC markers, such as ALDH1A1, CD44, and CD133. Tumors collected immediately after primary therapy were more densely composed of each marker, whereas samples collected at the first recurrence, before initiating secondary therapy, were composed of similar percentages of each marker as the primary tumor. These results suggest that stem cell subpopulations contribute to tumor chemoresistance and ultimately recurrent disease [20].

The enrichment of CSCs after chemotherapy can be simply explained by the remaining chemoresistant CSC population. However, recent evidence suggests that chemotherapeutic treatment results in the generation of ovarian CSCs. Emerging evidence is suggesting that epithelial-mesenchymal transition (EMT) plays a crucial role in chemoresistance and the generation of CSC populations [21–24]. The EMT and CSC-like cell phenotypes are closely related responses to chemotherapy [25]. Kurrey et al. reported that EMT-induced expression of the E-cadherin transcriptional repressors Snail and Slug has been shown to impose the acquisition of the CSC-like phenotype and chemoresistance in EOC cells by defying p53-mediated apoptosis [21]. Lafti et al. reported that cisplatin treatment of primary and metastatic EOC generates residual cells with mesenchymal stem cell-like profiles. The authors also demonstrated a significant enhancement in the sphere-forming abilities of ovarian cancer cells in response to chemotherapy drugs [19]. In addition, xenotransplantation studies using chemotherapy-treated EOC cells generated significantly larger tumor burdens compared with untreated cells, had a greater proliferative and tumorigenic capacity, and retained an enhanced stemness profile as evidenced by the enhanced expression of Ki67, CA125, CD117, and Oct4 [19].

## 3. Ovarian Cancer Stem Cell Model 

Many investigators have reported the identification and isolation of human ovarian CSCs based on either the differential expression of cell surface markers or differential biochemical properties. Accumulating evidence indicates that no single marker clearly identifies the ovarian CSC, and more recent reports have suggested that multiple cell populations defined by distinct marker profiles may in fact represent CSCs in EOC [26].

Since Bapat et al. first reported the isolation and identification of stem-like cells from the ascites of a patient with EOC [27], numerous putative cell surface and intracellular markers have been used to isolate and characterize ovarian CSCs. These include CD44, epithelial cell adhesion molecule (EpCAM), CD133, CD117, CD90 (Thy-1), CD24 [5, 28–31], and intracellular marker aldehyde dehydrogenase (ALDH) [32]. Ovarian CSCs can be successfully isolated via distinctive efflux of the DNA-binding dye Hoechst 33342. These ovarian CSCs are also called “side population” (SP) stem cells and have the capacity of self-renewal and differentiation in comparison with the non-SPs [33]. To date, the most commonly used markers for ovarian CSCs are CD133 and ALDH. ALDH (+) cells are inherently resistant to chemotherapy [34]. Small numbers of ALDH (+) cells can initiate tumors in mice, whereas a 10- to 50-fold excess of ALDH (−) cells cannot. Interestingly, cells that express both ALDH and CD133 possess greater tumor initiation capacity [34]. The identification of ovarian CSCs with various markers has been comprehensively reviewed [35, 36].

### 3.1. CSC Phenotypes and Metabolic Reprogramming

Although the metabolic requirements of CSCs are not fully understood, recent evidence suggests that pluripotent stem cells (PSCs) and cancer cell metabolism are overtly similar.

#### 3.1.1. Advantages of Aerobic Glycolysis: Pluripotency and Stemness

Briefly, rapidly proliferating non-CSC tumor cells are metabolically characterized by “aerobic glycolysis,” that is, high glycolysis even in the presence of oxygen, which provides tumor cells with 3 advantages: macromolecular biosynthesis, tumor invasion, and chemoresistance [37]. Recently, our group demonstrated that the overexpression of hexokinase II was an independent risk factor for chemoresistance using 111 EOC specimens [38]. In this study, hexokinase II overexpression was also found to be associated with short progression-free survival.

On the other hand, the glycolytic phenotype of CSCs is likely to be associated with pluripotency and stemness. Numerous studies have demonstrated that mouse and human embryonal stem cells (ESCs) and induced PSCs (iPSCs) exhibit elevated dependence on glycolysis under aerobic conditions compared with differentiated cells, such as cardiomyocytes and fibroblasts [39–42]. Somatic cells reprogrammed to pluripotency should become dependent on glycolysis [43]. During the reprogramming process, an increase in the expression of specific glycolytic genes precedes the gain of expression in genes that regulate self-renewal, suggesting that metabolic resetting has an early, active role in the return to pluripotency [44]. In addition, low oxygen content also helps to maintain ESC self-renewal and increases iPSC reprogramming efficiency. The parallel metabolic changes in oncogenesis and the induction of pluripotency elicit the hypothesis that cell bioenergetics can operate as the pivotal decision-making parameter during the reprogramming acquisition of stem cell properties in normal and non-CSC tumor cells [8]. In addition to the rapid energy production and the generation of building blocks, the glycolytic phenotype has a more fundamental role in inducing stemness. Therefore, it was suggested that the Warburg effect is the permitted cell metabotype possessing the necessary plasticity to reprogram the tumor cell of origin so that it can acquire the cellular state of a CSC [8].

The glycolytic phenotype appears to be closely associated with stemness. Liao et al. reported that spheroid cells, which were enriched for cells with cancer stem cell-like characteristics, routed glucose predominantly to anaerobic glycolysis and the pentose cycle to the detriment of rerouting glucose for anabolic purposes [45]. However, there is controversy over whether CSCs exhibit a glycolytic phenotype. The group of researchers supporting the “reverse Warburg effect” suggested that CSCs may rely on oxidative phosphorylation (OXPHOS) based on the selective toxicity of metformin on CSCs [46].

### 3.2. CSC and Tumor Microenvironment

The CSC model provides one explanation for the phenotypic and functional heterogeneity among cancer cells in EOC. The reversible CSC phenotype continuously evolves and can be switched on or off in response to cell-intrinsic or microenvironmental cues, including therapeutics and hypoxia [8, 47]. Additionally, molecular cues from stromal cells provide the signaling to maintain and expand the stem cell phenotype. An analysis of epithelial and mesenchymal markers in EOC reveals phenotypic heterogeneity and plasticity, and this phenotypic plasticity was dependent on external factors, such as stress created by starvation or contact with either epithelial or mesenchymal cells in cocultures [48]. Abelson et al. reported that putative ovarian CSCs with different levels of morphologic and tumorigenic differentiation display microenvironment-dependent plasticity with the capacity to restore self-renewal and CD44 expression [49]. In addition to the fact that ovarian CSCs can induce the formation of an inflammatory environment [50], stromal cells can also influence the maintenance of CSCs. A 4- to 8-fold increase in the percentage of putative ovarian CSCs has been observed in the presence of carcinoma-associated mesenchymal stem cells both in vitro and in vivo [51].

Conversely, CSCs can influence the tumor microenvironment. In xenotransplantation studies, which are the gold standard studies for CSC models, a relatively small number of transplanted CSCs must encounter a hostile microenvironment with hypoxia conditions and a lack of nutrients [52]. To promote tumor development, CSCs must have the ability to modulate the tumor microenvironment. Nevertheless, CSCs are supposed to have the ability to proliferate without preestablished microenvironmental support such as growth signals, inflammatory factors, or nutrients. Autonomous inflammatory activation and a preference for hypoxia should be the required abilities of CSCs.

#### 3.2.1. Inflammation and CSC

Ovarian CSCs induce inflammation in the microenvironment [50]. The chemoresistant subpopulation of EOC, through constitutive cytokine production, may significantly contribute to the maintenance of the inflammatory environment that promotes tissue repair and renewal [53]. The main trigger of constitutive nuclear factor kappa-light-chain-enhancer of activated B cells (NF-*κ*B)/cytokine production is IKK*β*, which is expressed only in the chemoresistant subpopulation [54]. Ovarian CSCs isolated from ascites and solid tumors are characterized by the following: CD44+, MyD88+, constitutive NF-*κ*B activity, cytokine and chemokine production, high capacity for repair, chemoresistance to conventional chemotherapies, resistance to TNF-*α*-mediated apoptosis, capacity to form spheroids in suspension, and ability to recapitulate in vivo the original tumor [5]. Constitutive NF-*κ*B activity and the secretion of proinflammatory cytokines have been demonstrated in the CD44+ population but not in CD44– EOC cells [5]. On the other hand, autocrine secretion of transforming growth factor- (TGF-*β*) by EOC cells has been shown to be responsible for an EMT-mediated increase of the CD44+/CD117+ population [55]. This effect was enhanced when EOC cells were cultured on fibronectin, demonstrating once again the additive effect of the different components of the tumor microenvironment on CSCs [56].

#### 3.2.2. Hypoxia and CSC

Hypoxia might be one of the key attributes in the tumor microenvironment that can regulate the phenotype of CSCs. Hypoxia was shown to maintain or upregulate ovarian CSC characteristics [57]. During hypoxia, anaerobic conditions prevail within the tumor, activating oncogenes such as* MYC *and* RAS*, resulting in the expression of hypoxia inducible factor- (HIF-) 1 and HIF-2 and the induction of the expression of pluripotent genes, such as* Oct4*,* Sox3*, and* kruppel-like factor- (KLF-) 4* [47, 57]. In another study, hypoxia facilitated the survival of CD117-enriched CSCs in EOC cell lines and cells derived from primary ovarian tumors through the activation of Wnt/beta-catenin and the ATP-binding cassette G2 downstream of CD117 [58]. Hypoxia has also been shown to induce EMT differentiation in CD44+ My88+ enriched ovarian CSC-derived xenografts obtained from primary ovarian tumors and ascites [59].

### 3.3. Plasticity of CSC

In contrast to the traditional view of a one-way CSC hierarchy in which CSCs give rise to non-CSCs but not vice versa, resulting in a hierarchical cell-lineage structure reflective of normal tissue biology [30, 60], the phenotypic plasticity of CSC was recently demonstrated. Gupta et al. recently reported that subpopulations of cells purified for a given phenotypic state from a breast cancer cell line return towards equilibrium proportions over time [61]. The authors proposed an alternative scenario in which there is bidirectional interconversion between CSC and non-CSC states [61].

Although many researchers have reported the clinical implication of targeting CSC surface markers, the fact that the ovarian CSC phenotype was not a consistent state but a changeable state depending on external conditions has been overlooked [36]. An emerging consensus in the field is that the cellular state rather than the cellular phenotype is crucial to defining and investigating CSCs [8]. However, the phenotypic plasticity of CSC and its dependence on the microenvironment do not imply that CSCs are illusive targets in cancer therapy but convince us that we must focus on where CSCs originate and how CSCs are generated and maintained under various conditions.

## 4. Metformin: A Promising Metabolic Approach Targeting CSCs in Ovarian Cancer

**Figure 1:**
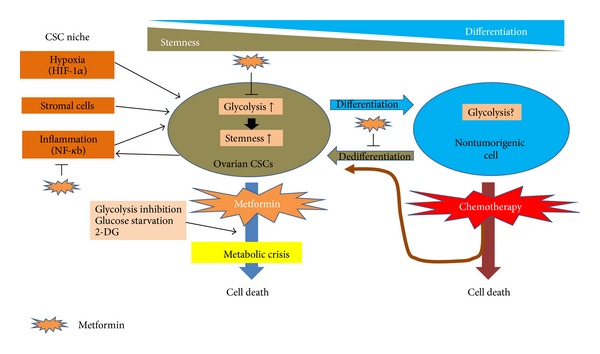
Mechanism of action of metformin in the concept of dynamic cancer stem cells in ovarian cancer. In contrast to the hierarchically organized cancer stem cell (CSC) model, the dynamic CSC model represents bidirectional interconversion between CSC and differentiated non-CSC states. The acquisition and maintenance of CSC characteristics are affected by microenvironmental cues, including inflammation, stromal cells and hypoxia, and therapeutics, such as chemotherapy. These factors eventually constitute the CSC niche (gray field). Hypoxia, which causes glycolysis, maintains and upregulates ovarian CSCs characteristics. Chemotherapy kills rapidly proliferating nontumorigenic cells, sparing chemoresistant CSCs. Chemotherapy also induces the acquisition of stem cell characteristics via epithelial-mesenchymal transition. Many studies using embryonal stem cells and induced pluripotent stem cells have demonstrated that glycolysis plays a fundamental role in inducing stemness. It is hypothesized that glycolysis may have a critical role in acquiring the CSC phenotype. It remains to be elucidated whether the metabotype is different between tumorigenic CSCs and rapidly proliferating nontumorigenic cells. Assumptive mechanisms of metformin's synergic effect on chemotherapy and selective toxicity to CSCs are illustrated. Metabolic stress caused by metformin may inhibit the transition to the glycolytic phenotype, resulting in the prevention of the acquisition of stemness and dedifferentiation. Metformin may also target the inflammatory components present in the tumor microenvironment. Ovarian CSCs may lack the ability to cope with metabolic stress caused by metformin and glucose starvation.

Many in vitroand in vivostudies have demonstrated the antiproliferative action of metformin on various cancer cell lines and animal cancer models (Table 1 and Figure 1) [13, 62–65]. This review will focus on metformin in detail.

### 4.1. Metformin on Cancer Stem Cells

The selective toxicity of metformin on CSC has been reported in various cancers. Selective inhibition of CSCs by metformin was first reported in 2009 in preclinical breast cancer models [11]. These results were subsequently extended to cancer cell lines from prostate and lung adenocarcinomas, where metformin similarly inhibited CSCs [68]. Recently, a selective toxicity on CSCs was also reported in EOC [13]. Metformin was shown to act on ovarian CSCs, reducing the percentage of ALDH (+) CSC in vitro and in vivo and inhibiting the growth of ovarian tumor spheres. Metformin was active against primary human ovarian CSCs in vitro, and metformin therapy alone slowed the growth of ovarian CSC in vivo [13].

Although the molecular mechanism by which metformin inhibits the self-renewal of CSCs is still obscure, it is noteworthy that iPSCs downregulate the expression of the catalytic subunit of the AMP-activated protein kinase (AMPK), which is a negative regulator for anabolic processes [69]. Activation of AMPK provides a metabolic barrier to reprogramming somatic cells into stem cells [70]. The AMPK activators established a metabolic barrier to reprogramming that could not be bypassed, even through p53 deficiency, a fundamental mechanism to greatly improve the efficiency of stem cell production. Monitoring the transcriptional activation status of each individual reprogramming factor (i.e., Oct4, Sox2, Klf4, and c-Myc) revealed that AMPK activation could prevent the transcriptional activation of Oct4, the master regulator of the pluripotent state. AMPK activation appears to impose a normalized metabolic flow away from the required proimmortalizing glycolysis that fuels the induction of stemness and pluripotency, endowing somatic cells with an energetic infrastructure that is protected against reprogramming. Decreased AMPK expression correlated significantly with higher tumor grade and was of adverse prognosis in EOC [71].

### 4.2. Clinical Evidence of Metformin in the Prevention and Treatment of EOC

A meta-analysis concluded that patients with diabetes exhibited a statistically significant increased risk of EOC [72]. Type 2 diabetic patients with EOC have poor survival outcomes and are more likely to exhibit poorly differentiated tumor histology compared with nondiabetic EOC patients [73]. These findings can be simply explained by the growth-promoting effect of chronic elevated plasma insulin levels and persistent elevated plasma glucose levels [74]. On the other hand, metformin has been demonstrated to have a chemopreventive and anticancer effect in many types of malignancies, including EOC [75, 76]. In a recent meta-analysis, metformin exhibited a tendency to reduce the occurrence of EOC among diabetic patients with the pooled odds ratio of 0.57 (95% confidence interval, 0.16–1.99) [77]. Furthermore, Kumar et al. reported in a case-control study that metformin intake was associated with a better survival in EOC [76]. Interestingly, metformin-associated tumors were more likely to be of lower grade and earlier stage and had a more favorable histology profile [76].

### 4.3. Mechanism of Action of Metformin in the Prevention and Treatment of EOC

#### 4.3.1. Insulin and IGF-1

**Figure 2:**
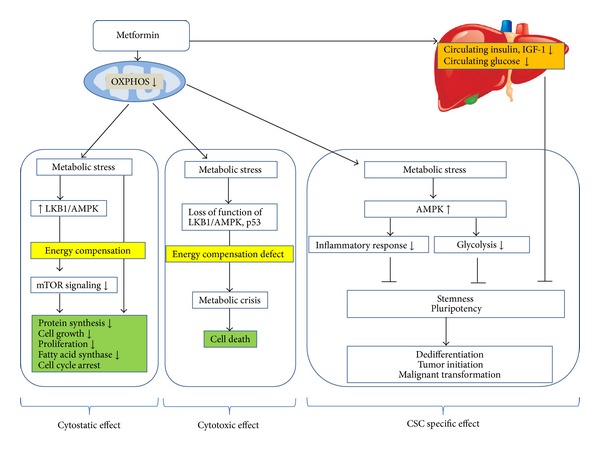
Antineoplastic mechanisms of action of metformin. The mitochondria are the primary target of metformin. Metformin interferes with oxidative phosphorylation via interactions with respiratory complex I, resulting in reduced ATP production and metabolic stress. Metformin lowers plasma glucose levels by decreasing gluconeogenesis and glucose uptake, resulting in lower circulating insulin and IGF-1 levels. An energy compensation reaction occurring in tumor cells capable of responding to metabolic stress is shown in the left box. By activating the LKB1/AMPK pathway, metformin inhibits mTOR downstream signaling, ultimately causing downregulation of energy-consuming processes and an overall cytostatic effect. The antitumour effects of metformin are regulated by both AMPK-dependent and AMPK-independent mechanisms. If tumor cells lack ability to cope with energetic stress due to the loss of function of LKB1/AMPK or p53, they may undergo a metabolic crisis leading to death (middle box). The right box presents an assumptive mechanism of metformin's action against CSCs. Both inflammation and the glycolytic phenotype are likely to be associated with pluripotency and stemness. The activation of AMPK provides a metabolic barrier to reprogramming somatic cells into stem cells. Metformin has been demonstrated to inhibit dedifferentiation processes, tumor initiation, and malignant transformation. Anti-inflammatory effects, restoration from glycolysis, and reduced growth signals might explain the anti-CSC action of metformin.

It has not been clearly confirmed how metformin decreases cancer incidence and prolongs the survival of cancer patients. A number of possible mechanisms regarding the anticancer effects of metformin have been suggested (Figure 2). The association between metformin and a reduced risk of cancer in diabetic patients may simply be explained through the action of metformin on the improvement in blood glucose and insulin levels because hyperinsulinemia and hyperglycemia play an important role in cancer proliferation [78, 79]. By decreasing the circulating levels of insulin and IGF-1, metformin may ameliorate this negative effect of hyperinsulinemia in diabetic patients.

#### 4.3.2. Metformin Induces Metabolic Stress

Metformin's primary activity is the inhibition of complex I of the mitochondrial electron-transport chain, resulting in an increase of the intracellular AMP/ADP ratio and thereby activating AMPK, a negative regulator of anabolic process (Figure 2) [80]. The inhibition of OXPHOS leads to lower ATP levels and reprogramming of cellular energy metabolism in favor of conserving energy and restoring ATP levels, ultimately causing downregulation of energy-consuming processes and an overall cytostatic effect [81]. Metformin appears to have a direct action on tumor growth both in vitroand in vivoby a mechanism involving the activation of the LKB1/AMPK pathway and the subsequent modulation of downstream pathways controlling cellular proliferation. The antineoplastic activity of metformin via AMPK activation is mediated through the inhibition of mTORC1 signaling, leading to inhibition of protein synthesis and cell proliferation [81]. mTOR plays a major role in carcinogenesis, and its activation is linked with cancer progression and poor outcomes in EOC [82].

Metformin induces metabolic stress by reducing mitochondrial ATP production. Therefore, it has been suggested that metformin could inhibit the growth of cancer cells by decreasing the cellular energy status and force a metabolic conversion that cancer cells are unable to execute [83]. A study revealed that AMPK activation promotes the survival of cells metabolically impaired by glucose limitation in part through p53 activation [84]. Moreover, loss of p53 impairs the ability of cancer cells to respond to metabolic changes induced by metformin and to survive under conditions of nutrient deprivation [85]. Furthermore, LKB1-deficient cells were more sensitive to metformin-induced energy stress when cultured at low glucose concentrations and were unable to compensate for the decreased cellular ATP concentration, causing cell death [86]. These cytotoxic effects of metformin arise only in the context of a genetic defect, such as loss of p53 and/or LKB1, that is present in the cancer but not in the normal host tissue, providing opportunities for “synthetic lethality” [81]. Glucose deprivation induces metabolic stress, resulting in AMPK activation. 2-Deoxyglucose (2DG), a well-known glycolysis inhibitor, can induce apoptosis in combination with metformin in various cancer cell lines, including EOC [87–89]. Cheong et al. reported that 2DG and metformin led to significant cell death associated with a decrease in cellular ATP, prolonged activation of AMPK, and sustained autophagy [88]. Importantly, forced energy restoration with methyl pyruvate reversed the cell death induced by 2DG and metformin, suggesting that prolonged activation of AMPK by 2DG and metformin might reflect sustained bioenergetic stress due to the failure of mitochondrial compensation.

Recent studies have revealed the existence of an alternative AMPK-independent pathway. Rattan et al. demonstrated that metformin treatment can arrest the cell cycle, decrease cyclin D1 expression, increase p21 protein expression, attenuate mTOR-S6 RP phosphorylation, and inhibit protein-translational and lipid biosynthetic pathways in EOC [66]. Although these antineoplastic effects of metformin coexisted with the activation of the LKB1/AMPK pathway, the effects were reproduced in AMPK-silenced EOC cells but not in LKB1 inactivated cells [66]. Therefore, it was suggested that LKB1 may have pivotal role in metformin's antineoplastic action. In fact, loss of the LKB1 and PTEN tumor suppressor genes in the ovarian surface epithelium was reported to induce papillary serous ovarian cancer [90]. Metformin induced apoptosis in EOC cell lines in an AMPK-independent manner and provoked a cell cycle arrest in the S and G2/M phase [65].

#### 4.3.3. Inflammation

Another potential mechanism is based on the positive impact of metformin on chronic inflammation. The role of chronic inflammation in promoting ovarian tumorigenesis and cancer progression has been well demonstrated elsewhere [91]. Metformin has been shown to decrease the production of inflammatory cytokines, including TNF-*α*, interleukin-6, and vascular endothelial growth factor, through the inactivation of NF-*κ*B and HIF-1*α* [92–94]. Emerging results demonstrating the capacity of AMPK to inhibit the inflammatory responses suggest that metformin may also target the inflammatory component present in the tumor microenvironment [95]. In addition, several reports demonstrated that metformin treatment inhibits neoplastic angiogenesis, resulting in the reduction of tumor growth [13, 64, 96, 97]. Wu et al. reported that metformin inhibits the development and metastasis of EOC by reducing neovascularization and macrophage infiltration [96].

#### 4.3.4. Reactive Oxygen Species (ROS)

Complex I inhibition is partially involved in metformin's growth inhibition of EOC, possibly by increasing ROS and sensitizing cancer to additional oxidative stress. Phenethyl isothiocyanate (PEITC) induces EOC cell death by increasing ROS. When given together, metformin and PEITC exhibit a synergistic increase in cell death in several EOC cell lines, including cisplatin-resistant cell lines [62].

### 4.4. Synergistic Anticancer Effects of Metformin and Chemotherapeutic Agents

Metformin has been demonstrated to augment the effects of various chemotherapeutic regimens by improving their efficacy as well as overcoming the chemoresistance in EOC (Table 1) [63–65, 67]. In fact, most in vitro studies used doses of metformin between 1 and 40 mM, which is well above the feasible therapeutic plasma levels (2.8–15 *μ*M) in humans [98]. Whereas the cytotoxic effect of metformin alone was achieved at millimolar concentrations in most studies, Erices et al. observed cytotoxicity with micromolar metformin in combination with chemotherapy at concentrations where the chemotherapy alone produced no loss in viability [63]. The exact mechanism of the synergetic effect of metformin on chemotherapy has not been well illustrated in most studies. One of the explanations is that metformin might be selectively toxic to CSC, which has been regarded as the cause of chemoresistance. As mentioned above, metformin could selectively target CSCs and act together with chemotherapy to block tumor growth and prolong remission in breast cancer [11, 68], pancreatic cancer [99], and EOC [13].

## 5. Conclusion

CSCs are believed to be one of the main causes of chemoresistance because CSCs are quiescent but possess clonogenicity on their own. Recent studies have demonstrated a dominant role for the tumor microenvironment in determining CSC characteristics within a malignancy. Although the metabolic phenotype of CSCs is not well defined and differs significantly between types of cancer, metformin is thought to represent an emerging lethal weapon against CSCs in ovarian cancer on the basis of the capability to control the CSC niche as well as metabolic modulation. Nevertheless, there seems to be much to be elucidated regarding better characterization of CSCs as well as the interaction between CSC and the CSC niche moleculogenetically and metabolically. Further research of metformin is urgently required for effectively overcoming the chemoresistance of EOC by selectively targeting the metabolic features of ovarian CSCs.

## Figures and Tables

**Table 1:** Preclinical studies of metformin targeting metabolism of ovarian cancer cells and/or ovarian CSCs.

Agent or drug (dosage)	CSCs enriched	Alone	Combination with other drugs	Mechanism of action	Reference
Metformin (0.02 mmol/L in vitro)	No	No significant loss of viability or change in cell cycle	Improvement of cytotoxic response to carboplatin	<—>	[63]

Metformin (5 mmol/L in vitro), PEITC (5 *µ*mol/L in vitro)	No	Inhibition of growth in vitro	Combination with PEITC increases cell death in vitro	ROS generation	[62]

Metformin (5 mmol/L in vitro)	No	Induced apoptosis in vitro	Combination with cisplatin enhances apoptosis	AMPK-independent, downregulating Bcl-2/Bcl-xL, upregulating Bax/Bad	[65]

Metformin (100–200 mg/kg in vivo)	No	Inhibition of ovarian tumor growth, proliferation, metastasis, and angiogenesis in vivo	Combination with cisplatin reduces tumor growth	AMPK/mTOR, antiangiogenic effect	[64]

Metformin (5 mmol/L in vitro)	No	Inhibition of proliferation in vitro	<—>	Cell cycle arrest, AMPK/mTOR and AMPK independent pathway	[66]

Metformin (5–50 mmol/L in vitro)	No	Inhibition of proliferation in vitro	Improvement of cytotoxic response to cisplatin	AMPK/mTOR	[67]

Metformin (0.3 mmol/L in vitro, 150 mg/kg in vivo)	ALDH+ cells	Inhibition of ovarian CSC/TIC growth in vitro, nonsignificant decreases in tumor growth in vivo	Combination with cisplatin restricts tumor growth in vivo	<—>	[13]

CSC, cancer stem cell; ROS, reactive oxygen species; AMPK, adenosine monophosphate-activated protein kinase; mTOR, mammalian target of rapamycin; ALDH, aldehyde dehydrogenase; PEITC, phenethyl isothiocyanate; TIC, tumor initiating cell. Modified and adapted with permission from reference [15].
